# Characterization of Staphylococcal Cassette Chromosome *mec* Elements from Methicillin-Resistant *Staphylococcus pseudintermedius* Infections in Australian Animals

**DOI:** 10.1128/mSphere.00491-18

**Published:** 2018-11-07

**Authors:** Kate A. Worthing, Sybille Schwendener, Vincent Perreten, Sugiyono Saputra, Geoffrey W. Coombs, Stanley Pang, Mark R. Davies, Sam Abraham, Darren J. Trott, Jacqueline M. Norris

**Affiliations:** a Sydney School of Veterinary Science, University of Sydney, NSW, Australia; b Department of Microbiology and Immunology, The University of Melbourne at the Peter Doherty Institute for Infection and Immunity, VIC, Australia; c Institute of Veterinary Bacteriology, University of Bern, Bern, Switzerland; d Australian Centre for Antimicrobial Resistance Ecology, School of Animal and Veterinary Sciences, University of Adelaide, Roseworthy, SA, Australia; e Research Center for Biology, Indonesian Institute of Sciences, Bogor, Indonesia; f Australia Antimicrobial Resistance and Infectious Diseases Laboratory, School of Veterinary Life Sciences, Murdoch University, Murdoch, WA, Australia; g PathWest Laboratory Medicine—WA, Fiona Stanley Hospital, Murdoch, WA, Australia; University of Nebraska Medical Center

**Keywords:** SCC*mec*, *Staphylococcus pseudintermedius*, antimicrobial resistance, methicillin resistance, veterinary, zoonotic infections

## Abstract

We examined the oxacillin resistance phenotype and genomic structure of staphylococcal cassette chromosome *mec* (SCC*mec*) elements from 77 veterinary methicillin-resistant Staphylococcus pseudintermedius (MRSP) isolates. Isolates were characterized by oxacillin broth microdilution, whole-genome sequencing, and bioformatics analysis. Five previously described SCC*mec* elements, and a sixth novel element, were identified: SCC*mec* III (also known as II-III), ΨSCC*mec*_57395_, and SCC*mec*_NA45_ (a SCC*mec* VII variant), all previously described in MRSP, and SCC*mec* IVg and SCC*mec* V_T_, previously described in both methicillin-resistant Staphylococcus aureus (MRSA) and MRSP. The sixth element was novel and found among nine geographically clustered isolates. This novel pseudostaphylococcal cassette chromosome (ΨSCC*mec*_KW21_) contained a class A *mec* gene complex but lacked *ccr* genes. It also harbored heavy metal (cadmium) resistance determinants. The median oxacillin MIC values among ΨSCC*mec*_KW21_, SCC*mec* III, and SCC*mec* V_T_ isolates were significantly higher than those determined for the SCC*mec*_NA45_ VII variant isolates and ΨSCC*mec*_57395_ and SCC*mec* IVg isolates. ΨSCC*mec*_KW21_ was found exclusively in sequence type 497 (ST497), an MRSP clone that is locally successful in Victoria, Australia. Future studies are necessary to determine if this clone has disseminated further afield and if ΨSCC*mec*_KW21_ has moved into other MRSP lineages or staphylococcal species.

**IMPORTANCE** Staphylococcus pseudintermedius is a significant veterinary pathogen and occasional cause of infections in humans. β-Lactams are an important group of antimicrobials used to treat staphylococcal infections in humans and animals. However, when staphylococci become methicillin resistant via the acquisition of a mobile genetic element called staphylococcal cassette chromosome *mec* (SCC*mec*), they become resistant to all β-lactams. This study detected a novel SCC*mec* element among a cluster of methicillin-resistant S. pseudintermedius isolates from animals in Australia. It also detected SCC*mec* elements in S. pseudintermedius that had high similarity to those identified in methicillin-resistant Staphylococcus aureus, demonstrating how human and animal pathogens can share the same resistance determinants.

## Abstract (precis)

Staphylococcus pseudintermedius is a significant veterinary pathogen and occasional cause of infections in humans. β-Lactams are an important group of antimicrobials used to treat staphylococcal infections in humans and animals. However, when staphylococci become methicillin resistant via the acquisition of a mobile genetic element called staphylococcal cassette chromosome *mec* (SCC*mec*), they become resistant to all β-lactams. This study detected a novel SCC*mec* element among a cluster of methicillin-resistant S. pseudintermedius isolates from animals in Australia. It also detected SCC*mec* elements in S. pseudintermedius that had high similarity to those identified in methicillin-resistant Staphylococcus aureus, demonstrating how human and animal pathogens can share the same resistance determinants.

## OBSERVATION

Methicillin-resistant Staphylococcus pseudintermedius (MRSP) has become an important opportunistic pathogen in veterinary small-animal practice (1) and is an occasional zoonotic pathogen (2). S. pseudintermedius is part of the canine skin microbiota but can cause a wide range of opportunistic clinical infections across many body systems. MRSP infections are more complicated than methicillin-susceptible S. pseudintermedius (MSSP) infections due to the lack of potential treatment options. In staphylococci, methicillin resistance is determined by the *mecA* gene and its homologues, *mecB* and *mecC* (3–5). *mecA* and *mecC* are harbored within a mobile genetic element called the staphylococcal cassette chromosome *mec* (SCC*mec*) element (4, 6), whereas *mecB*, typically found in Macrococcus caseolyticus (7), has recently been detected on a multidrug resistance plasmid in methicillin-resistant Staphylococcus aureus (MRSA) (5). SCC*mec* elements are composed of a methicillin resistance determinant (*mecA, mecB,* or *mecC*) contained within a *mec* gene complex and include site-specific recombinase genes (*ccr*), which are responsible for insertion of the SCC*mec* cassette into the core genome (6). SCC*mec* types were initially defined by their combination of the *mec* gene class complex and the *ccr* gene complex (8). However, assignment of nomenclature and classification of SCC*mec* elements have been hampered by the existence of composite cassettes (9, 10) and pseudo-SCC*mec* elements that do not harbor *ccr* genes (11). Eleven SCC*mec* cassettes are formally recognized in the database of the International Working Group on the Staphylococcal Cassette Chromosome (IWG-SCC) and were named I to XI according to the chronological order in when they were first reported. Several SCC*mec* elements are reported in MRSP, including SCC*mec* III (previously described as II-III [12]), which is found in the globally dominant sequence type 71 (ST71) lineage (13); SCC*mec* V_T_ variants (14); and a newly reported cassette, SCC*mec*_NA45_, which harbors a class C1 *mec* gene complex and *ccrC* recombinase gene (15). Other SCC*mec* elements have been identified in S. pseudintermedius that are not recorded in the IWG-SCC database, including ΨSCC*mec*_57395_ (11), which lacks *ccr* genes, and SCC*mec*_KM241_ (12) and SCC*mec*_AI16_ (10) (Table 1). Despite the difficulties associated with characterizing some SCC*mec* elements, SCC*mec* typing can provide useful information about the phylogenetic and epidemiological origin of isolates. Echoing the epidemiological division of human-derived MRSA into health care-associated (HA) and community-associated (CA) lineages (16, 17), Kasai et al. found that veterinary MRSP isolates with SCC*mec* III tended to be HA-MRSP lineages whereas isolates with SCC*mec* V tended to be CA-MRSP (18). Recently, we described the molecular epidemiology of 77 MRSP isolates collected from clinical infections in animals in Australia during a national surveillance study and found that the population was phylogenetically diverse (19). The current study characterized the SCC*mec* elements in these isolates.

**TABLE 1:** SCC*mec* elements previously identified in methicillin-resistant *S. pseudintermedius* isolates that are not described in the SCC*mec* database of the IWG-SCC

SCC*mec* name	Reference isolate	Accession number	*mec* complex	*ccr* complex
ΨSCC*mec*_57395_	MRSP 57395	HE984157.2	C1	No *ccr*
SCC*mec*_KM241_	MRSP KM241	AM904731.1	A	A5B3
SCC*mec*_AI16_	MRSP AI16	LN864705.1	A	A1B3
SCC*mec*_NA45_	MRSP NA45	CP016072.1	C1	C6

### Characterization of SCC*mec* elements.

Isolates originated from a larger collection of 669 S. pseudintermedius isolates collected during the first Australian survey into antimicrobial resistance in veterinary pathogens (20, 21). All isolates were identified using a BD Bruker MALDI Biotyper and were screened for methicillin resistance using manual oxacillin broth microdilution according to CLSI guidelines (22). The end dilution for MIC testing was 64 mg/liter oxacillin. Oxacillin resistance was confirmed by detection of an oxacillin MIC of ≥0.5 mg/liter using Vitek 2, in accordance with the manufacturer’s instructions, and detection of the *mecA* gene by whole-genome sequencing as previously described (19). DNA extraction, library preparation, and initial *de novo* assembly were undertaken as previously described (19). SCC*mec* typing was undertaken by downloading the sequences of the *mec* gene complex and *ccr* elements of the SCC*mec* elements described by the IWG-SCC (8) from the NCBI online database (https://www.ncbi.nlm.nih.gov/). SCC*mec* elements previously identified in S. pseudintermedius but not included in the *SCCmec* working group website were also downloaded (Table 1). Downloaded SCC*mec* element sequences underwent BLASTn searches against *de novo* contigs using CLC Genomics Workbench. BLASTn results required more than 85% sequence homology to be assigned a particular *ccr* gene. *mec* gene complexes were assigned based on the gene structure of *mecA*, its regulators, and associated insertion sequences (ISs). If a SCC*mec* type could not be assigned, contigs were mapped against a scaffold of reference SCC*mec* types (8) and the reference methicillin-susceptible S. pseudintermedius genome, ED99 (accession no. CP002478.1). The Kruskal-Wallis test was used to determine whether the median oxacillin MICs differed across SCC*mec* types. The Mann-Whitney U test was used to assess differences between SCC*mec* types in the median oxacillin MIC. SCC*mec* types with more than eight isolates were compared as separate entities in analyses; other isolates were grouped together.

### Diversity of SCC*mec* types.

Six SCC*mec* types were identified among 74 of the 77 MRSP from Australia (Table 2). The SCC*mec* type of the remaining three isolates (two ST497 isolates and one ST71 isolate) could not be determined because of poor sequencing quality (low read coverage and contig breaks) in the region around the *mecA* gene. Isolates from the same multilocus sequence type (MLST) tended to harbor the same SCC*mec* type. Four of the SCC*mec* types have previously been characterized in MRSP or MRSA as follows: SCC*mec* III (*n* = 34) and ΨSCC*mec*_57395_ (*n* = 7), previously described in MRSP; and SCC*mec* V_T_ (*n* = 10) and SCC*mec* IVg (*n* = 5), previously described in MRSP and MRSA (11, 12, 23, 24). The 34 SCC*mec* III isolates, which were mostly ST71 and ST316, demonstrated 98% to 100% sequence homology to the *mec* and *ccr* gene complexes of the III element from MRSP KM1381 (12). ΨSCC*mec*_57395_ isolates had no *ccr* genes but had 99 to 100% sequence homology to the region spanning from *orfx* to IS*256* from MRSP 57395 (11). The V_T_ isolates displayed 96% to 100% homology to the *ccrC1* gene from MRSP 06-3228 (23) and 100% homology to its *mecA* and IS*431* genes, but the *mecR1* gene was variably truncated from 23 bp to 93 bp. SCC*mec* IVg isolates had 97% to 100% sequence homology to the entire cassette from MRSA isolated from bovine milk described previously by Kwon et al. (24).

**TABLE 2:** SCC*mec* types and multilocus sequence types (MLST) of methicillin-resistant *Staphylococcus pseudintermedius* isolates from clinical infections in animals in Australia, 2013 to 2014

Element	MLST^*a*^
SCC*mec* III (*n*= 34)	ST71 (*n*= 25), ST316 (*n*= 8), ST25 (*n*= 1)
ΨSCC*mec*_57395_ (*n*= 7)	ST45 (*n*= 6), ST544 (*n*= 1)
SCC*mec* V_T_ (*n*= 10)	ST496 (*n*= 8), ST64 (*n*= 1), ST751 (*n*= 1)
SCC*mec* IVg (*n*= 5)	ST498 (*n*= 3), ST258 (*n*= 1), ST539 (*n*= 1)
SCC*mec*_NA45_ (VII variant^*b*^) (*n*= 9)	ST64 (*n*= 2), ST84 (*n*= 1), ST283 (*n*= 1), ST499 (*n*= 1), ST500 (*n*= 1), ST501 (*n*= 1), ST525 (*n*= 1), ST547 (*n*= 1)
ΨSCC*mec*_KW21_ (*n*= 9)	ST497 (*n*= 9)
Not determined^*c*^ (*n*= 3)	ST497 (*n*= 2), ST71 (*n* = 1)

a MLST, multilocus sequence types.

b The SCC*mec*_NA45_ VII variant element harbors a C1 *mec* complex and a *ccr*C6 element (15). ΨSCC*mec*_KW21_ is a novel element that is described in this paper.

c The SCC*mec* elements of three isolates could not be determined due to poor sequence and assembly quality (contig breaks around the *mecA* gene).

The fifth SCC*mec* element has 99% sequence homology to the novel element recently reported in MRSP NA45 (15). In our study, this element was identified in nine geographically dispersed isolates from eight different STs (Table 2). These isolates harbored a class C1 *mec* complex with 99% sequence homology to SCC*mec* X from MRSA JCSC6945 but did not harbor the same *ccr* genes as this element. Instead, the isolates harbored a recombinase gene with 99% homology to *ccrC6*, recently identified in a 43,922-bp SCC*mec* element in ST84 MRSP (15) and methicillin-resistant *S. schleiferi* (MRSS) (25). This element is also present in ST398 MRSA RIVM3897 (26), but the RIVM3987 element lacks the final 8,164 bp at the 3′ end of the SCC*mec* cassette in ST84 MRSP and MRSS. The nine isolates in this study showed 99% homology to the entire SCC*mec* cassette from MRSP NA45, which contained heavy metal resistance genes *arsB, arsC*, *arsR* (arsenic resistance) and *copA* (copper resistance) but no antimicrobial resistance genes. On the basis of typing recommendations of the IWG-SCC (8), the element in these nine isolates and in MRSP NA45 could be described as SCC*mec* VII because it harbors a class C1 *mec* gene complex and *ccrC* recombinase gene (Fig. S1). However, as the *mec* gene complex is positioned in reverse orientation in comparison to SCC*mec* VII, we feel that it is more appropriate to refer to this cassette as “SCC*mec*_NA45,_” a SCC*mec* VII variant. Most of the isolates harboring the SCC*mec* VII variant SCC*mec*_NA45_ did not harbor the variable repeat region of the *spa* gene and therefore could not be assigned a *spa* type (19).

10.1128/mSphere.00491-18.1 FIG S1 Alignment of the SCC*mec* cassette from MRSP isolate NA45 (15), found in nine isolates in this study. The cassette is aligned with SCC*mec* X and VII from MRSA. The NA45 cassette shows high homology to the class C1 *mec* gene complex of SCC*mec* X and >90% homology to the *ccrC* gene of SCC*mec* VII, but the orientation of the *mec* gene complex is reversed between NA45 and SCC*mec* VII. The alignment was produced in EasyFig V.5 (34). Download FIG S1, TIF file, 2.7 MB. Copyright © 2018 Worthing et al. 2018 Worthing et al. This content is distributed under the terms of the Creative Commons Attribution 4.0 International license.

The final nine MRSP isolates, all ST497, could not be mapped to previously described SCC*mec* elements. All ST497 isolates were from a geographic cluster in Melbourne, Victoria (19). To characterize the novel element found in the remaining nine isolates, a representative ST497 isolate (KW21) underwent further sequencing by Illumina HiSeq and Nanopore long-read technology using MinIon. MinIon long reads were used to verify the structure of the *de novo* assembly. *De novo* assembly of Illumina HiSeq reads using Geneious yielded a 319,216-bp contig that contained the entire putative SCC*mec* element. This contig was subjected to a blast search against the NCBI database to determine putative components of the element. The element was annotated using PROKKA (27) and manually verified using the BLASTn algorithm in CLC Genomics Workbench.

### ΨSCC*mec*_KW21_ element.

**FIG 1:**
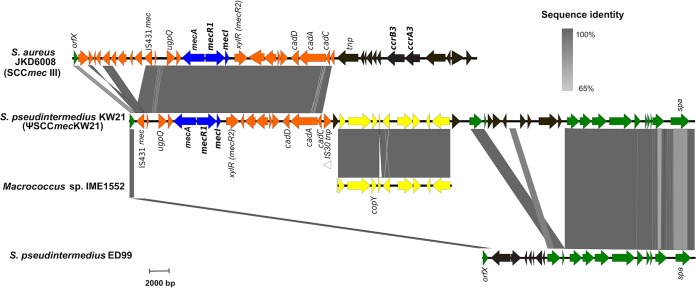
Alignment of the novel SCC*mec* element ΨSCC*mec*_KW21_, its flanking regions, and related sequences. The 16,711-bp ΨSCC*mec*_KW21_ element, delineated by *orfX* and a truncated IS*30* transposase, is a truncated version of SCC*mec* III. The *mecA* gene complex and cadmium resistance genes (*cad*CAD) of ΨSCC*mec*_KW21_ have high homology the same region in ST239 MRSA (S. aureus JKD6008). ΨSCC*mec*_KW21_ inserts into the genome of KW21 at the 3′ end of the *orfX* gene and is bordered by an ∼10,000-bp region with 99% homology to *Macrococcus* sp. IME1552. This region, which includes a putative copper transport repressor (*cop*Y), is then bordered by sequence with 99% homology to the region harboring the *spa* gene in methicillin-susceptible S. pseudintermedius ED99. Areas of sequence homology, determined by BLASTn, are indicated with gray shading. Green arrows, coding DNA sequences (CDS) shared with MSSP ED99; orange arrows, CDS shared with S. aureus JKD6008; blue arrows, class A *mec* gene complex; yellow arrows, CDS shared with *Macrococcus* IME1152; black arrows, unique CDS. The alignment was created in EasyFig V.5 (34).

The novel SCC*mec* element, designated “ΨSCC*mec*_KW21_,” was integrated at the 3′ end of the *orfX* gene (*rmlH*) (Fig. 1). The characteristic direct repeat and insertion sequences (ISs) that typically flank SCC*mec* elements were absent at the right side (10, 28). The element contained a class A *mec* gene complex that had 99% BLASTn similarity to the class A *mec* gene complex SCC*mec* cassettes from MRSA JKD6008 (29), MRSP KM241 (12) and MRSP AI16 (10) (accession numbers CP002120.1, AM904731.1, and LN864705 respectively). The element did not contain any *ccr* genes. The 16,711-bp region of ΨSCC*mec*_KW21_ that spanned from *orfX* to the cadmium resistance operon, *cadCAD,* had 99% sequence homology to the same region of SCC*mec* III from ST239 MRSA isolate JKD6008 (Fig. 1). We therefore propose that ΨSCC*mec*_KW21_ is a truncated version of SCC*mec* III that lost the segment containing *ccr* genes after the cassette was inserted into the genome. Unlike SCC*mec* III from ST239 MRSA, ΨSCC*mec*_KW21_ does not harbor *ccr* genes; instead, *cadA* and *cadC* are bordered by a truncated IS*30* family transposase. To the right of this transposase is a 10,116-bp region that had 97% BLASTn similarity to a chromosomal region of *Macrococcus* sp. IME1152 (accession number CP017156.1) that includes the *cop*Y gene, encoding a putative copper transport repressor. BLASTn analysis of the *de novo* contigs of ST497 isolates against the SCC*mec* element from KW21 revealed that all nine isolates had the same *mec* gene complex, the genomic region with *Macrococcus* sequence homology, and no *ccr* genes. Consequently, they were considered to have the same pseudoelement as KW21. ST239 is an important health care-associated MRSA lineage in humans, so screening of healthy and diseased animals across Australia is now indicated to determine if ST497 and/or other lineages harboring ΨSCC*mec*_KW21_ continue to exist within a geographic cluster or whether this lineage has disseminated across Australia or overseas, as has occurred with ST239 MRSA in humans (30).

### Variation in oxacillin MICs amongst different SCC*mec* types.

**FIG 2:**
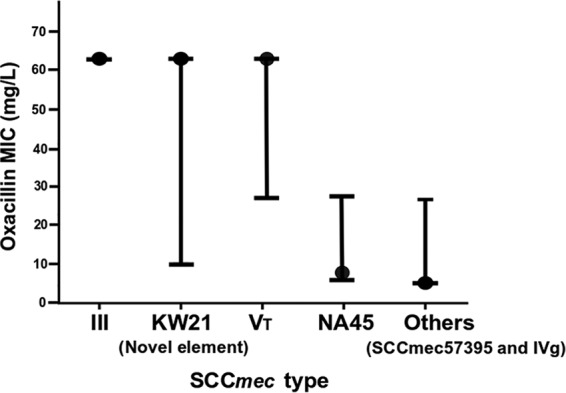
Oxacillin MIC for SCC*mec* types of MRSP isolates from Australia collected in 2013 to 2014. Black dots indicate the median MIC value; error bars indicate the interquartile range.

Fig. 2 shows the oxacillin MIC range for the major SCC*mec* types. The median oxacillin MICs differed significantly depending on SCC*mec* type (Fig. 2; *P* < 0.001). The median oxacillin MICs of ΨSCC*mec*_57395_ and SCC*mec* IVg isolates (1 mg/liter) and of SCC*mec*_NA45_-VII variant isolates (4 mg/liter) were significantly lower than the median oxacillin MIC of SCC*mec* III, SCC*mec* V_T_ and ΨSCC*mec*_KW21_ isolates (64 mg/liter; *P* < 0.01). Recently, Kasai and colleagues (18) similarly reported differences in oxacillin MIC on the basis of MRSP SCC*mec* types. Specifically, they found that isolates with SCC*mec* III generally had higher oxacillin MICs and were more often associated with suspected hospital-acquired infections than isolates with SCC*mec* V. Analogous to analyses of MRSA in human medicine, they concluded that oxacillin MIC may give clues as to an isolate’s epidemiological origin, where a high oxacillin MIC may indicate that an MRSP isolate is from a successful “health care-associated” clone whereas isolates with lower MICs may represent “community-associated” clones (18). There were insufficient epidemiological data available in our study to draw similar conclusions, but our results do support the notion that the oxacillin MIC is significantly affected by the SCC*mec* type and that isolates of the same multilocus sequence type generally harbor the same SCC*mec* type. Thus, it follows that different MRSP lineages would demonstrate different oxacillin MICs. The *mecA* gene can be repressed by either *mecI* or *blaI*, but MRSA and MRSP isolates with *bla* regulators typically demonstrate more rapid induction and higher expression of *mecA* than isolates with *mec* regulators alone (31–33). To determine whether the oxacillin MIC was influenced by the presence of the *blaI* and *mecI* genes rather than simply by the SCC*mec* type, we screened all isolates for these repressor genes using a BLAST-based command line script (screen_assembly3.py: https://github.com/shimbalama/screen_assembly). Most (45/77) isolates harbored *blaI,* but only SCC*mec* III and ΨSCC*mec*_KW21_ harbored both *blaI* and *mecI.* The high median oxacillin MIC (64 mg/liter) of III and ΨSCC*mec*_KW21_ isolates could have been due to the fact that *blaI* attenuates the strong *mecA* repression expected from the cognate *mec* regulators (32).

In summary, we found six SCC*mec* types among 77 MRSP isolates collected from clinical infections in Australian animals. The oxacillin MIC varied according to SCC*mec* type. We also described ΨSCC*mec*_KW21_, a novel pseudo-SCC*mec* element that was found only in a geographic cluster of clinical isolates. This report highlights the utility of nation-wide surveillance studies in unearthing novel and emerging resistance determinants and demonstrates how genomic resistance elements found in significant human pathogens such as S. aureus can also be found in veterinary pathogens such as S. pseudintermedius.

### Accession number(s).

The genomic sequence of ΨSCC*mec*_KW21_ has been deposited in the National Center for Biotechnology Information (NCBI) database under GenBank accession number MH713898. The contig sequences of the MRSP isolates described in this study were also deposited under BioProject number PRJNA439160 and BioSample accession numbers SAMN08741522 to SAMN08741590.
