# Supplementary figures and images for: Characterization of Staphylococcal Cassette Chromosome mec Elements from Methicillin-Resistant Staphylococcus pseudintermedius Infections in Australian Animals
Source: mSphere. 2018 Nov 7;3(6):e00491-18. doi: 10.1128/mSphere.00491-18 (PMC6222048; doi:10.1128/mSphere.00491-18)

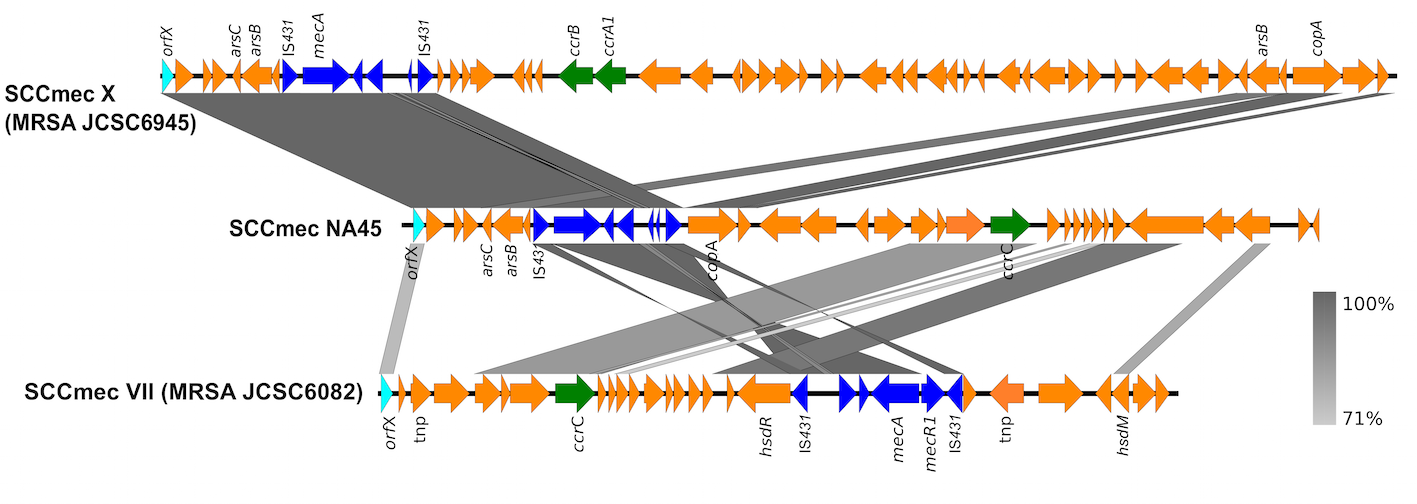

Supplement: FIG S1 [file sph006182698sf1.tif]
